# Increasing pneumococcal vaccine uptake in older adults: a scoping review of interventions in high-income countries

**DOI:** 10.1186/s12877-022-03653-9

**Published:** 2023-01-02

**Authors:** Abirami Kirubarajan, Meghan Lynch, Sharifa Nasreen, Gebremedhin B. Gebretekle, Shaza A. Fadel, Natasha S. Crowcroft, Sara Allin

**Affiliations:** 1 Temerty Faculty of Medicine, University of Toronto, Toronto, Canada; 2 Institute of Health Policy Management and Evaluation, University of Toronto, Toronto, Canada; 3 Centre for Vaccine Preventable Diseases, Dalla Lana School of Public Health, University of Toronto, Toronto, Canada; 4 Toronto Health Economics and Technology Assessment (THETA) Collaborative, University Health Network, Toronto, Canada

**Keywords:** Pneumococcal, Vaccine, Senior, Uptake, Older adults, Vaccine hesitancy

## Abstract

**Background:**

There is low uptake of the pneumococcal vaccination in eligible older adults, even in high-income countries that offer routine and universal vaccination programs.

**Objective:**

To systematically characterize interventions aimed at improving pneumococcal vaccine uptake in older adults.

**Design:**

We conducted a scoping review following PRISMA-SCr guidelines of five interdisciplinary databases: Medline-Ovid, Embase, CINAHL, PsychInfo, and Cochrane Library. Databases were searched from January 2015 until April 2020. The interventions were summarized into three pillars according to the European Union Conceptional Framework for Action: information campaigns, prioritization of vaccination schemes, and primary care interventions.

**Results:**

Our scoping review included 39 studies that summarized interventions related to pneumococcal vaccine uptake for older adults, encompassing 2,481,887 study participants (945 healthcare providers and 2,480,942 older adults) across seven countries. Examples of interventions that were associated with increased pneumococcal vaccination rate included periodic health examinations, reminders and decision-making tools built into electronic medical records, inpatient vaccination protocols, preventative health checklists, and multimodal educational interventions. When comparing the three pillars, prioiritization of vaccination schemes had the highest evidence for improved rates of vaccination (*n* = 14 studies), followed by primary care interventions (*n* = 8 studies), then information campaigns (*n* = 5 studies).

**Conclusion:**

Several promising interventions were associated with improved outcomes related to vaccine uptake, although controlled study designs are needed to determine which interventions are most effective.

**Supplementary Information:**

The online version contains supplementary material available at 10.1186/s12877-022-03653-9.

## Introduction

The pneumococcal vaccine has been recommended in older adults to prevent illnesses caused by the *Streptococcus pneumoniae* bacterium, including invasive pneumococcal disease (IPD) and community-acquired pneumonia (CAP) [1–3]. In high-income countries, both IPD and CAP are leading causes of morbidity and mortality in older adults with underlying medical conditions, and contribute significantly to hospitalization rates [4]. Though vaccination guidelines differ internationally, the pneumococcal polysaccharide vaccine (PPV) is typically recommended for all healthy adults 65 years and older, as well as individuals at higher risk of disease (including individuals with a smoking history, experiencing homelessness, or substance use disorders) [5–7]. Currently licensed PPV vaccination offers protection against 23 strains of *S. pneumoniae* that account for approximately nine out of ten cases of pneumococcal disease in high-income countries [8]. Systematic reviews and meta-analyses have noted that pneumococcal vaccines are highly effective in preventing hospitalization, infection, and death in older populations [9–12].

However, even in high-income countries that offer routine and universal vaccination programs, there are concerns regarding uptake [13–15]. For example, a recent national survey conducted by the Government of Canada estimated that only 42.0% of eligible older adults have ever been vaccinated against pneumococcal disease [14]. Consequently, as part of the Global Vaccine Action Plan, has aimed to reach an 80% vaccination coverage rate for adults 65 years or older by 2025 through both policy and community interventions [15]. Similarly, an American study of Medicare claims from 2013 to 2015 identified that the majority of study patients did not receive a pneumococcal vaccine in the first year after turning 65 [13]. However, to our knowledge a systematic search of the literature has not been conducted regarding the best interventions to improve PPV uptake in older adults. Given the barriers for older populations to access preventative health interventions, we believed that it would be useful to summarize the available evidence of existing interventions for policymakers, researchers, and clinicians.

As such, we conducted a scoping review to systematically characterize and assess interventions to improve pneumococcal vaccine uptake in older adults.

## Methods

### Systematic search

The scoping review was conducted according to the standards and guidelines established in the Preferred Reporting Items for Systematic Reviews and Meta-Analysis with extension for Scoping Reviews (PRISMA-ScR), and was informed by the scoping review framework proposed by Arksey & O’Malley [16, 17].

We conducted a systematic literature search of five interdisciplinary databases: Medline-Ovid, Embase, CINAHL, PsychInfo, and Cochrane Library. Keywords and Medical Subject Headings were related to our population (i.e.; older adults over the age of 65) and the intervention (i.e., pneumococcal vaccine). Databases were searched from January 2015 until April 2020. The cut-off date was selected based on preliminary searches of the literature and the feasibility of the research design, and to obtain recent literature on the topic. Prior to running our search, we consulted a University of Toronto librarian with public health expertise for feedback on the search strategy.

Eligibility criteria are outlined in Table 1. Studies that reported outcomes specific to older adults (defined as average age of 65 years and above, or self-describing as including older adults or synonyms such as “elderly patients”, or “seniors”) in high-income countries (based on the World Bank classification of countries by income) were eligible for inclusion [18]. Articles relevant to conditions requiring pneumococcal vaccination (e.g., asplenia, HIV) with a median age of less than 65 or not self-described as being specific to older adults were not eligible. Interventional study designs (e.g., randomized controlled trials, pilot projects, quality improvement projects) as well as cohort or cross-sectional designs that defined an intervention as the exposure (e.g., before and after a policy intervention) were eligible for inclusion. To capture emerging trends in the research, conference papers and abstracts were eligible for analysis. We did not include grey literature or websites. The search was limited to articles published in the English language and available as full-texts online. Deduplication and study import was done via the Covidence platform [19].

**Table 1:** Eligibility Criteria

Population: Older adults (typically defined as over the age of 65, all studies with median age of over 65 or self-describing as relevant to older adults were included)
Intervention: Any intervention (e.g. educational campaigns, primary care changes, guidelines)
Comparator: Any comparator (e.g. no intervention, standard of care, another intervention)
Outcomes: Any outcome reported in the literature (e.g. vaccinations rates, clinical outcomes, awareness or education-related outcomes, implementation outcomes)

### Study selection, extraction, and analysis

Prior to screening, a pilot study using a standard pilot extraction form was performed on 15 random articles (5 qualitative, 5 mixed-methods, and 5 quantitative studies) by two team members involved in the screening process. The pilot extraction form included the following information: article title, article authors, full abstract, include/exclude/maybe, reasons, and other (for any noteworthy information that did not fit in the previous categories). Each team member independently screened the 15 articles and then a group discussion was held to compare findings. The reasons for inclusion and exclusion were discussed and discrepancies were resolved through discussions with additional team members.

Following the pilot, study screening for both titles and abstracts was completed in duplicate by at least two independent reviewers. A group of three authors reviewed articles in the first stage, and a group of authors reviewed articles in the second stage. All discrepancies were resolved via consensus between the three authors. The Covidence platform was used for database screening [19].

The data extraction for each article was performed in duplicate by at least two independent reviewers. Prior to the final data extraction, the authors completed a pilot extraction and modified the data extraction form based on emerging themes in the articles. Items for data extraction included study design, year of publication, intervention type, characteristics of study population, and key outcomes. Outcomes were summarized descriptively via thematic analysis, as due to the heterogeneous nature of the targeted population, intervention, outcome, study design, settings, and methods. Thematic analysis was determined via consensus approach based on the key findings and the conclusions of the included literature. During extraction, key interventions were organized into the three pillars of the Conceptional Framework for Action to Improve Vaccine Coverage, including (1) information campaigns, (2) prioritization of vaccination schemes, and (3) primary care interventions [20, 21]. The Conceptional Framework for Action is outlined in Table 2. If interventions were relevant to more than one pillar, the most relevant pillar was decided via consensus approach among the lead authors (i.e. discussion-based agreement) for the purposes of categorization, though the outcomes for all pillars were summarized. Outcomes of interest included vaccinations rates, clinical outcomes (e.g., pneumococcal infections or hospitalizations), awareness or education-related outcomes, and implementation outcomes such as provider feedback or cost-effectiveness.

**Table 2:** Conceptional Framework for Action to Improve Vaccination Coverage

Pillar	Description	Examples
I Information campaigns	Dissemination of educational materials regarding vaccination, including advantages, indications, protections, dosing, and logistics	●Personalized communication campaigns ●Training interventions for healthcare providers ●Mass communication campaigns (e.g. publications, television, radio)
II Prioritization of vaccination schemes	Decision-making tools that support the priority vaccination of select groups based on the available evidence, and practical resources that enable this prioritization	●Decision-making toolkits, checklists, protocols, and algorithms ●Universal funding ●Policy for distribution and prioritization
III Primary care-based interventions	Interventions housed within primary care (e.g. generalist care, family medicine) that take leverage existing relationships and familiar infrastructure to promote vaccination	●Reminder systems for family physicians ●Financial incentives for family physicians ●Bundling of vaccines for convenience​ ●Encouraging healthcare providers to engage in vaccine discussions that are non-confrontational and participatory​

Methodological quality for individual studies was graded by a single reviewer using an adapted rating scale based on the Cochrane Risk of Bias (ROB) and Risk of Bias Assessment tool for Non-randomized Studies (RoBANS) tools [22, 23]. For the risk of bias assessments, the criterion related to blinding was removed from the assessment tool due to the lack of applicability to the interventions.

## Results

### Search yield

**Fig. 1:**
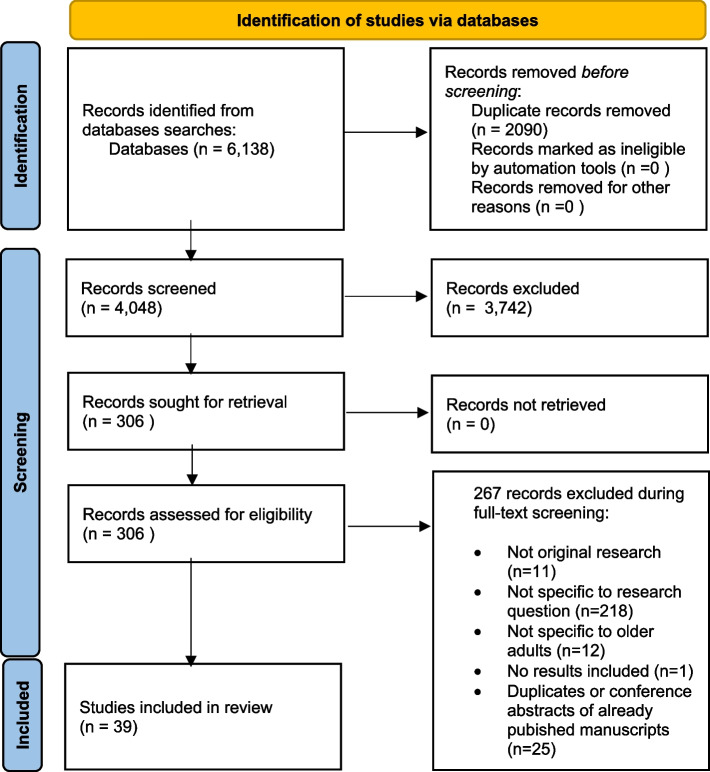
PRISMA Diagram

Results of the study screening process are available in the PRISMA diagram in Fig. 1. The search yielded 6138 studies, with 2090 as duplicates. A total of 4048 unique studies were screened for title and abstract screening, with 306 meeting eligibility and proceeding to full-text screening. Of the 306 full-text articles screened, a total of 39 studies, including nine abstracts, were included in the review [24–62].

### Article characteristics

Details of individual studies are available in Supplementary Tables 1 and a summary of key findings are available in Table 3.

**Table 3:** Summary of Key Results

	Sample Size (%)
Total number of participants	2,481,887
Number of healthcare workers	945 (0.3%)
Number of older adults	2,480,9422,481,887 (99.7%)
Number of studies included	*n* = 39
Study Location	
United States	30/39 (76.9%)
Singapore	3/39 (7.7%)
Japan	2/39 (5.1%)
Canada	1/39 (2.6%)
Hong Kong	1/39 (2.6%)
Australia	1/39 (2.6%)
Germany	1/39 (2.6%)
Study Design	
Interventional	34/39 (87.2%)
Controlled	15/34 (44.1%)
Randomized	5/34 (14.7%)
Observational	5/39 (12.8%)
Type of pneumococcal vaccine	
PPV23	7/39 (17.9%)
PVC13	1/39 (2.6%)
Both PVC13 and PPV23	14/39 (35.8%)
Outcome Measures	
Vaccination rates	*n* = 34
Information or awareness scores	*n* = 9
Implementation outcomes	*n* = 6
Pillar according to European Union Conceptional Framework for Action	
Information campaigns	*n* = 15
Prioritization of vaccination schemes	*n* = 14
Primary care interventions	*n* = 10

There was a total of 2,481,887 study participants (945 healthcare providers and 2,480,942 older adults) across the 39 studies. The majority of studies were located in the United States (*n* = 30), with the remaining studies located in Singapore (*n* = 3), Japan (*n* = 2), Canada (*n* = 1), Hong Kong (*n* = 1), Australia (*n* = 1), and Germany (*n* = 1).

A total of 34 studies were interventional designs, fifteen of which employed a control group and four of which were randomized. Of the interventional studies, two articles were pilot studies and eight were quality improvement projects. The remaining five studies were retrospective cohort or time series or followup population studies that evaluated intervention-related exposures (e.g., enhanced primary care services) or larger policy changes, rather than classical interventional studies. These studies evaluated whether a specific intervention (e.g., periodic health examinations) were associated with higher rates of vaccination.

While most of the studies were specific to the general older adult population (*n* = 25), the remainder targeted older adults from special populations such as Indigenous populations (*n* = 1) or African American/Black populations (*n* = 1), hospitalized or internal medicine patients (*n* = 6), residents at long-term care homes (*n* = 1), veterans or military personnel (*n* = 2), or individuals with chronic diseases, including leukemia and chronic kidney disease (*n* = 3). Eighteen articles were not specific to a type of pneumococcal vaccine, seven articles specifically examined PPV23, one article specifically examined PVC13, and the remainder examined both PVC13 and PPV23 subtypes.

The studies described diverse outcomes, including vaccination rates (*n* = 34), information or awareness scores (*n* = 9), and implementation outcomes, including cost-effectiveness (*n* = 6). Duration of follow-up ranged from two weeks to five years.

The majority of studies were rated as medium risk of bias (*n* = 20), with ten studies rated as low risk of bias (Supplementary Table 2). All nine studies rated as high risk of bias were peer-reviewed abstracts as they did not meet minimal standards for formal risk of bias assessment.

### Analysis according to the European Union Conceptional Framework for Action

#### Information campaigns

Fifteen studies [26, 28, 30, 32–36, 39, 40, 46, 51, 53, 59, 62] evaluated the effect of educational interventions on pneumococcal vaccination awareness, uptake, and clinical outcomes. Seven studies evaluated educational interventions aimed at healthcare providers (e.g., family physicians, internal medicine residents, nurses), while the remainder examined the impact on older patients, including patients with chronic diseases. Educational interventions were multimodal, including video-based interventions, visual pamphlets and flashcards, skits with actors, visual abstracts, telephone-based counselling, and in-person education. Five interventions were pharmacist-led, while two interventions were nurse-led.

Eight studies noted that these interventions significantly improved awareness and knowledge related to pneumococcal vaccination, including eligibility, dosing schedules, and benefits of immunization. Specifically, one study noted the most improvement of knowledge in older adults with lower socioeconomic status or education [34]. In addition, five studies noted that educational interventions modestly improved pneumococcal vaccination rates in eligible older adults, with absolute differences ranging from 0.6% to 9.0% in comparison to control. Notably, one studydemonstrated that educational interventions for both healthcare professionals and older adults increased vaccination by 3.9% across 400 practice sites. 27 This intervention corresponded to a clinical outcome of a significant reduction in pneumococcal disease of 3 cases per 10,000, as well as less frequent hospitalization and inpatient mortality. Of note, this was the only study that reported disease as an outcome. Implementation-related considerations included the importance of information available in multiple languages, [34] as well as materials without redundancy or contradictions [28]. Another study reported that the average program cost for vaccine education was $119.00 USD, and their intervention resulted in 37.2% of unvaccinated participants reporting receiving the vaccine by the 3 month mark [51].

#### Prioritization of vaccination schemes

Prioritization of vaccination schemes (i.e., systemic decision-making, algorithms, and protocols) was evaluated in fifteen studies [29, 31, 41, 44, 45, 47–50, 52, 54–58]. Four studies targeted under-vaccinated or at-risk populations, including Indigenous populations [57], military hospitals [29], oncology units [31], long-term care facilities [54], and inpatient units [41]. Fourteen studies demonstrated improved rates of vaccination in older populations, as well as improved administration of the correct pneumococcal vaccines to the appropriate patient. These prioritization interventions included pharmacist-led distribution schemes, standing orders for nurses to administer vaccines and appropriate algorithms/protocols for distribution, improved protocols for eligibility (e.g. notification within electronic medical record when accessing a patient’s chart), standardized checklist guidelines), and routine vaccination guidelines on a national level. Benefits of prioritization of vaccination schemes included convenient workflow, removal of practical barriers (i.e. administrative supports or personnel), as well as standardization of care. In addition, four studies noted that the interprofessional lens of prioritization (e.g., through hiring of dedicated pharmacist teams improved the quality of care. The longest follow-up was completed by Patel et al. (2018) through a 5-year retrospective cohort and statistical modelling, which noted that pharmacy-led teams in the United States increased national immunizations by an estimated 3.5 million doses per year [49].

However, one retrospective cohort study conducted in Australian Indigenous populations noted a decline in vaccination rates (from 30.0% in 2004–2005 to 23.5% in 2012–2013) in those aged 50–64 but there were no significant changes in those aged over 65 [57]. This trend occurred despite national prioritization of vaccines through a universal funding scheme. The same study noted that pneumococcal coverage in remote areas significantly decreased in 50–70 year-olds. There were unclear reasons for this unexpected result, although the authors highlighted the need for collaboration with Indigenous stakeholders, as well as innovative solutions aimed at remote areas. Another study that created a pharmacy-led initiative (comprising of online modules aimed at optimizing pharmacists’ role in vaccination, marketing tools, and a systematic protocol for screening) found no significant differences overall between intervention and baseline groups for pneumococcal vaccination (*p* = 0.134), although there may have been a slight increase when examining only the last 6 months of the intervention.

Two studies evaluated cost-effectiveness for prioritization. One study noted that estimated cost per patient was an increase of $40.43 USD after incorporating pharmacy members in the screening and selection process for vaccination, yielding an average of 55.0% increase in vaccination [50]. Another study (Wells 2019) noted that the return on investment for a pharmacy resident-driven vaccination campaign was 29.2%, as the total revenue and cost generated by the protocol over the 60-day period was $8276.10 and $6406.88 USD, respectively. 57.

None of the prioritization-related studies reported clinical outcomes (e.g., infection rates, hospitalization, mortality) related to pneumococcal disease.

#### Primary care interventions

Eleven studies evaluated interventions based within primary care [24, 25, 27, 31, 37, 38, 42, 43, 60, 61, 63]. Eight studies noted significant improvement in vaccination rates. For example, one prospective before-and-after non-randomized trial decreased missed opportunities for pneumococcal vaccinations by 6.6% in the intervention group versus 4.3% in the comparator group (*p* < 0.0001), through a multimodal primary care successful intervention, such as standing orders for vaccination, provider reminders, and performance reports.42 Another study conducted a retrospective cohort of 10,318 Medicare beneficiaries to note that patients with a periodic health examination were significantly likely to obtain pneumococcal vaccination.36 Other successful interventions included primary care reminder systems, periodic health evaluations, primary care decision-making support, as well as interprofessional teams within family medicine (including physicians, nurses, and pharmacists).

However, two studies noted no significant improvements with provider reminders, quarterly provider-level performance reports, or enhanced primary care models [38, 60]. Hurley et al. (2019) noted that barriers to a centralized vaccine reminder system were the lack of availability of appointments as well as the prioritization of the influenza vaccine over pneumococcal or tetanus vaccines [38]. In contrast, Zimmerman et al. (2016) conducted a randomized controlled cluster trial of a four-pillar program (including improved convenience of scheduling, communication with family medicine patients, enhanced office systems, and motivation through an in-office immunization champion) noted no difference in vaccination in intervention groups during the first year of intervention, and mixed results in the second year depending on site (i.e. in small or medium-sized private primary care practices, there were significant increases in pneumococcal vaccination while there were no differences in larger preventative practices) [60].

None of the studies reported clinical outcomes related to pneumococcal disease.

## Discussion

Our scoping review included 39 studies that summarized interventions related to pneumococcal vaccine uptake for older adults, encompassing approximately 2.5 million participants across seven countries. The majority of studies were rated as low or medium risk of bias (*n* = 29), as ten abstracts were rated as high risk of bias. We summarized the available interventions into three pillars according to the European Union Conceptional Framework for Action: information campaigns, prioritization of vaccination schemes, and primary care interventions. Outcomes included awareness or knowledge among patients and implementation-related measures such as cost-effectiveness or stakeholder feedback. Many interventions improved outcomes related to vaccination uptake (such as awareness, acceptability, and/or vaccination rate) in eligible older adults. Examples of interventions that were associated with increased pneumococcal vaccination rate included: periodic health examinations, reminders and decision-making tools built into electronic medical records, inpatient vaccination protocols, standing orders for nurses, preventative health checklists, and multimodal educational interventions.

However, it is difficult to directly compare interventions across studies (for example, on the basis of intervention setting or target group) due to the heterogeneity of the contexts, designs, and results. Ultimately, stronger study designs such as randomized controlled trials that directly compare different interventions (i.e. in contrast to evaluating multiple interventions simultaneously) are needed to determine which interventions are most effective. In addition, it is important to note that only one study examined clinical outcomes and found that infection rates decreased due to the studied interventions. As this is the most clinically relevant outcome, further studies should consider directly evaluating reduction of disease burden (including morbidity and mortality) in their study design.

Overall, our review aligns well with current literature in public health. Previous studies related varicella zoster virus (VZV) vaccination, COVID-19, and influenza vaccines have found that there is a strong need for improved vaccine awareness among at-risk populations [63–65]. However, awareness alone is often not enough to improve vaccination rates or clinical outcomes. Previous systematic reviews have documented the myriad of logistical barriers for patients to access other public health interventions (e.g. cancer screening, harm reduction programs), such as scheduling concerns, prioritization within the health visit, or lack of dedicated personnel [66–68]. As such, the successful interventions described in this scoping review provided diverse options beyond education-based strategies, ranging from reminder systems to convenient scheduling to dedicated immunization staff. The included studies also considered interprofessional perspectives, including pharmacists, nurses, primary care physicians, and hospitalist physicians, as there are multiple opportunities for a patient to receive vaccination throughout their medical care. It is also important to note that collaborative approach is often required between the disciplines, as there may be diffusion of responsibility between healthcare providers. For example, hospitalist physicians may believe that vaccination is the role of a family physician, while the family physician may believe that the patient has already been served during their in-hospital stay. Therefore, it is important for the available interventions to involve communication between stakeholders as well as feature transparent role distribution.

The included studies demonstrated that there is a need for increased health services and implementation research related to vaccine uptake, particularly related to improving coverage. Although pneumococcal disease is responsible for tens of thousands of deaths per year, vaccination rates have not reflected the severity of the disease [69]. While there were several studies with high quality designs (e.g., controlled or randomized designs, multimodal interventions, dedicated patient research prior to implementation, appropriate length of follow-up), the majority of our studies evaluated a single intervention and did not feature reproducible or comprehensive designs. Future research should also consider the diverse outcomes that are required to determine whether an intervention should be implemented. For example, several studies had a focus solely on vaccine-related awareness, but not on whether patients actually decided to receive the vaccine. However, for many patients, awareness is not the only limiting factor regarding vaccine uptake. For example, administrative and financial barriers also pose concern for patients. As such, prior to the implementation and evaluation of an intervention, it is important to gather patient perspectives and address barriers accordingly.

Future research should continue to target specific high-risk groups for under-vaccination, such as individuals experiencing homelessness, chronic illness, or mental illness, and those from refugee and immigrant groups [70–72]. It is also important to note that racialized individuals also face unique barriers to vaccination, such as discrimination and subsequent mistrust of the medical system [73, 74]. Although public health interventions should aim to serve the greatest number of patients as possible, future interventions may wish to focus on the individuals who are most vulnerable for IPD and CAP. Our review found promising interventions that focused on African American/Black populations [51], military hospitals [29], oncology units [31], and inpatient units [41]. However, these studies stressed the importance of continued efforts to serve marginalized populations and address underlying causes for vaccine hesitancy.

Most pressingly, this research has become increasingly relevant due to the ongoing COVID-19 pandemic [75]. The pandemic has highlighted the need to address vaccine hesitancy in older adults, especially community-based adults and those who have been marginalized from health systems. Many older adults have COVID-19 vaccine hesitancy or uptake barriers, with reasons including distrust of the health system, inability to answer relevant questions, or administrative barriers [76–78]. Many of the recent studies that have aimed to address this issue have featured similar interventions to the ones outlined in this scoping review, including reminders built into electronic medical record, discharge reminder tools, and educational initiatives [79–81]. As such, the findings of this scoping review may be relevant in the idea generation and design of interventions aimed at improving COVID-19-vaccine uptake among older adults.

Strengths of our scoping review include our rigorous search of five interdisciplinary databases, as well as our robust screening and data extraction in duplicate. We consulted a public health librarian prior to running our search, and considered broad search criteria in order to improve the sensitivity of our strategy. The scoping review design was selected in order to capture a wide range of heterogenous studies, as well as identify knowledge gaps in an emerging evidence base. Our data was analyzed according to a relevant and appropriate framework that has been used internationally in conceptualizing public health interventions. In addition, our scoping review is particularly timely given COVID-19 vaccine hesitancy among older adults during the ongoing pandemic. Nonetheless, our review has several limitations. Namely, our conclusions may not be generalizable as several studies in the scoping review were low quality designs. However, we conducted a robust quality assessment in this scoping review to mitigate this potential weakness. Although the majority of our full-text studies were evaluated as low to medium risk of bias, there is a need for reproducible study designs that feature control groups, adequate length of follow-up, as well as comprehensive outcomes reporting. Finally, as with all scoping reviews, there is a potential for studies to have been missed in this review, particularly as titles/abstracts may not have specifically identified as relevant to older adults. In addition, we only included articles in the English language and did not search grey literature. There may also be limitations to inter-rater agreement due to our multiple extractors. However, we have taken multiple steps to minimize these limitations through our broad search terms in consultation with a research librarian, in addition to our multiple stages of screening.

Ultimately, our review features several promising options for improving pneumococcal vaccine uptake among older adults, as outlined above. However, it is important to note that not all interventions may be suitable or reproducible in different clinical or population contexts. As such, clinicians and policymakers who are interested in improving vaccine uptake in older adults may wish to combine aspects of the summarized interventions and adapt interventions according to their community’s unique needs.

## Conclusion

Our scoping review included 39 studies that summarized interventions related to pneumococcal vaccine uptake for older adults. We synthesized the available interventions into three pillars according to the European Union Conceptional Framework for Action: information campaigns, prioritization of vaccination schemes, and primary care interventions. When comparing the three pillars, prioiritization of vaccination schemes had the highest evidence for improved rates of vaccination (*n* = 14 studies), followed by primary care interventions (*n* = 8 studies), then information campaigns (*n* = 5 studies). Several promising interventions were associated with improved outcomes related to vaccine uptake for eligible older adults, including periodic health examinations, reminders and decision-making tools built into electronic medical records, inpatient vaccination protocols, preventative health checklists, and multimodal educational tools. However, stronger study designs such as randomized controlled trials are needed to determine which interventions are most effective. Specifically, future studies should evaluate the cost-effectiveness of interventions and the impact on clinical outcomes (such as morbidity and mortality), as this was understudied throughout the included articles.

## Supplementary Information

**Additional file 1:** **Supplementary Table 1.** Details of individual studies. **Supplementary Table 2.** Risk of Bias Assessment – Cochrane ROB and Cochrane RoBANs.

## Data Availability

All data generated or analysed during this study are included in this published article.
