# Supplementary material for: Increasing pneumococcal vaccine uptake in older adults: a scoping review of interventions in high-income countries
Source: BMC Geriatr. 2023 Jan 2;23:2. doi: 10.1186/s12877-022-03653-9 (PMC9807101; doi:10.1186/s12877-022-03653-9)
Supplement: Supplementary file 1 — Additional file 1: Supplementary Table 1. Details of individual studies. Supplementary Table 2. Risk of Bias Assessment – Cochrane ROB and Cochrane RoBANs. [file 12877_2022_3653_MOESM1_ESM.docx]

Supplementary Table 1: Details of individual studies

| **Pillar** | **Citation, Country** | **Details of Study**  **(e.g. design, population)** | **Sample Size and Population** | **Details of Intervention** | **Key Outcomes** |
| --- | --- | --- | --- | --- | --- |
| I. Information campaigns | Brown et al. (2016), USA | Assessment of patient education video via focus groups of 23 participants as well as assessment from 73 participants | 99 older adults (23 from focus group, 73 participants in final stage) | Patient education video promoting pneumococcal vaccine | The video was well-received by patients (i.e. easy to understand, informative). |
|  | Caffrey et al. (2017), USA | Multi-center study with pre-post design – pre-intervention phase was 1 year, intervention phase was two years, and post-intervention phase was two years | 380 immunization providers | Pharmacist-driven academic detailing and community outreach, involving distribution of vaccine-related materials | Pneumococcal vaccination rate increased significantly (absolute difference 3.9%, percentage change in proportion 5.4%; p = 0.01), pneumococcal disease decreased (decrease of 2.74 per 10,000 cases, p = 0.02) |
|  | Chan et al., (2015), Hong Kong | Cluster randomized controlled trial with clusters within individual weeks over a 10 week period – 2517 patients with chronic diseases | 2517 older adults with chronic diseases | Nurse-delivered brief muti-component educational tool (3 min telephone intervention, 3 minute face-to-face intervention, heath educational leaflets and/or video) | Vaccination rate was higher in the intervention group (57% vs 48%; relative risk = 1.20), but there was no difference in awareness at 3-month follow-up (65% vs 59%, relative risk = 0.86) |
|  | DeCarlo et al (2018), USA | Interventional quality improvement design | Patients aged over 65 at a Geriatrics Clinic | Multimodal with physician education, training other healthcare providers (MAs, LPNs, RNs) and optimizing EMR | Increased from 66.% to 67.7% (no p value) |
|  | Gladman et al (2016), USA | Interventional quality improvement design with repeat chart reviews and 4 and 8 months after implementation | 3591 patients aged over 65 in a large academic general internal medicine practice | Visual abstract for clinic nurses that summarizes pneumococcal vaccination recommendations, accompagnied by standing orders and nurse education | Increased from 33% to 43% in less than one year (no p value reported) |
|  | Ho et al. (2016), Singapore | Quality improvement project for 124 older adults, no follow-up specified, no control group | 124 older adults | Short survey about knowledge, attitudes and beliefs, accompanied by educational brief and invitation for vaccination | 63.6% of older adults were willing to receive of the vaccination following the educational brief. |
|  | Ho et al. (2017), Singapore | Mixed-methods study with focus group discussion, and then an educational intervention aimed at 604 older adults | 32 older adults | Educational intervention in multiple languages with both visual and written materials | Median knowledge scores improved from 5 out of 9 (IQR 4-5) to 7 out of 9 (IQR 6-8) following the intervention. |
|  | Ho et al. (2019), Singapore | Pragmatic, cluster-randomized controlled trial of adults over the age of 65 | 4378 participants in intervention arm and 4459 in control arm | Point-of-care informational intervention in general practitioner clinics | Pneumococcal vaccination uptake rates were higher during the intervention period compared with the control period (5.7% vs 3.7%; p = .001) |
|  | Jolin et al., (2016), USA | Multiple quality improvement initiatives of 16 IM residents, measured pre/post intervention  Special population of interest was was veterans who were discharged from hospital medicine service | 16 internal medical residents | Nursing, pharmacist, and physician education, automated reminders, edited discharge summaries | Vaccine uptake increased from 30 to 87% for veterans discharged from hospital (no test of significance was reported). |
|  | Kawczak et al., (2020), USA | Quality improvement project with intervention and control cohorts, measured via vaccination receipts | 100 primary care physicains; 361,528 patient reeipts | Variety of learning interventions (e.g., guidelines reviews, online educational modules) and teaching regarding action planning, all aimed at providers | There was a significant increase in pneumococcal vaccination (80.6% pre-intervention vs. 82.7% post-intervention; p < 0.001) for those > 65 years of age. |
|  | McRae et al. (2016), USA | Pre-post intervention with 3 months follow-up of 203 older adults | 203 older adults | Pharmacist-led multimodal educational intervention for patients, including formal presentation, live skit, small group action planning, and optional vaccination offer | There were significant improvement (numeric results were not specified) in mean belief scores from baseline regarding the vaccine as well as pharmacists as the ones providing the vaccine. |
|  | Pizzi et al (2018), USA | Interventional design with three months of follow-up | 190 older adults (mean age 74.3, SD 8.9), a majority of whom were African American/Black men (80.5%) | Educational initiative with pharmacist presentation, actors' kit and small-group action planning | Knowledge increased by 54.2% on assessment questionnaires at the three-month mark. Program cost was $119.00 USD per attendee. 37.2% of unvaccinated participants reporting receiving the vaccine by the 3 month mark. |
|  | Ratner et al (2016), USA | Interventional quality improvement design with pre- and post-comparison | Unspecificed number of primary care patients over the age 65 | Patient reminders via mail, phone calls, or electronic medical record, with social workers assisting with logistical barriers such as transportation. | Vaccination increased from 64.4% to 77.5% (no p value). |
|  | Wilson et al (2017), USA | Interventional quality improvement design measuring pre- and post-intervention vaccination rates and knowledge | 101 internal medicine residents, number of older adults not reported | Multicomponent educational curriculum aimed at internal medicine residents | Vaccination rates increased from 18.5% to 39.0% (p=0.0001). After the intervention, quiz scores increased for each item and were statitically significant for four out of eight items (no p values reported, no values reported). |
|  | Zhao et al (2017), USA | Interventional study with prospective, one-tailed design | 55 older adults over the age of 60 | Education initaitive with face-to-face individual communication for patients | Increased vaccination rates (pre vs. post) were observed for PPSV23 (53.3% vs. 68.9%, p = 0.016) and PCV13 (15.6% vs. 43.8%, p = 0.004). |
| II. Prioritization of vaccination schemes | Carillo et al. (2017), USA | Pilot immunization initiative at a large primary care service, uncontrolled | 3054 pre-intervention, 2922 patients post-intervention | Military clinical pharmacists and technicians were used to identify eligible individuals, provide vaccine, and educate | In the post-intervention period, there were higher rates of pneumococcal vaccination for individuals who had not previously received any vaccines (6.5 to 22.6%, no p value reported) |
|  | King, Judd et al. (2017), USA | Assessment of pharmacist led intervention, controlled via pre-policy cohort and post-policy cohort | 100 patients in the pre-policy stage, 45 post-policy stage | Pharmacist-led intervention in which inpatients were assessed for pneumococcal vaccine administration alongside streamlined electronic orders. | Vaccine rates for the correct formula (on the basis of age or medical condition) increased from 40% to 95.6% |
|  | McAdam-Marx et al. (2020), USA | Retrospective controlled study between 2013 and 2017 with longitudinal adherence analysis | 12,057 older adults in baseline period; 14,518 older adults in interim period; 16,689 older adults in follow-up period | Pneumococcal-specific best practice alerts (i.e. notification within electronic medical record when accessing a patient’s chart) with and without workflow design | There was no difference in vaccination rates for the best practice alerts with or without workflow redesign. |
|  | McKeirnan and Sarchet et al. (2019), USA | Interventional design (not controlled, not randomized) 1 year of follow-up | 7 pharmacy technicians (administration of 427 pneumococcal vaccines) | Implementation of Pharmacy Technician immunization Program to employ specially trained pharmacists to administer a range of vaccines | Improved vaccination uptake (a total of 427 pneumococcal vaccines administered). |
|  | Mendu et al. (2014), USA | Prospective study with pre/post intervention | 13 primary care providers – 7 control, 4 interventional; 105 patients with chronic kidney disease for control and 263 for intervention | Implementation of a preventative health checklist for healthcare providers related to chronic kidney disease, with pneumococcal vaccine as one of the items | Patients in the intervention group were significantly more likely to have received pneumococcal vaccination (27.8% for control, 65.7% for intervention, p=0.001). |
|  | Naito et al. (2020), Japan | Analysis of cumulative number of vaccines shipped to each municipality, divided by population aged over 65 years | Unspecified number of over the age of 65 who are eligible for vaccination | National vaccination program by Japanese government, involving vaccination subsidies and routine vaccination | Vaccination rate increased to 40.6% two years into the 5-year national immunization campaign, with significant (unspecified) increases from the baseline for each fo the previously studied periods (exact numbers not available for baseline) <p=0.01) |
|  | Patel et al., (2018) USA | Assessment of national immunization data following a major American policy change | 1,446,453 respondents, of which 431,183 were above 65 years of age | Policy change to allow pharmacy-based immunization services | Pharmacy-based immunization services was attributed to an additional 3.5 million additional pneumococcal vaccinations. |
|  | Pickren et al. (2016), USA | Retrospective cohort study that examined pneumococcal rates in a control group versus intervention | 272 patients | Pharmacy provided screening and selection for vaccines in eligible individuals | The compliance to CDC guidelines for vaccination increased from 42% to 97%. The intervention group had increased labor cost for assessments and vaccine-related expenditure. |
|  | Rager et al. (2016), USA | Interventional design with randomized pharmacy stores in a 2:1 ratio of test stores and control stores | 196 patients (either older adults or patients with chronic conditions) with a mean age of 66 (SD 13), 403 providers (169 pharmacists and 234 technicians) | Online modules aimed at optimizing pharmacists' role in vaccination, marketing tools, and screening | There was no overall change in pneumococcal vaccination rate (n=1383 in baseline, n=449 in control; rates were then averaged per month) between baseline and intervention groups (p=0.134). |
|  | Shittu et al. (2020), USA | Quality Improvement design based on Diffusion of Innovation theory | 112 residents at a long-term care facility (average age of 83) | Nursing education, standing order protocol | Pneumococcal immunization rates increased from 56% pre-intervention to 82% post-intervention, and vaccination screening rates increased from 0% to 100%. A chi-squared test for independence indicated a significant relationship between vaccination status and implementation of the standing order protocol intervention (p=0.046, df=1, n=112). |
|  | Shono et al. (2019), Japan | Cross-sectional survey to assess vaccination rates, uncontrolled but considered variables such as coverage | 3899 respondents | Routine vaccination program that included subsidization of vaccine across the entire country | Eligibility for subsidy had the largest effect on vaccination uptake (adjusted odds ratio: 16.7) in second year of 5-year rollout. |
|  | Turbeville et al. (2018), USA | Quality Improvement design based on Diffusion of Innovation theory, data collected electronically by information technology specialists and designed as pre- and post-intervention | 353 patients at an outpatient internal medicine clinic, aged over 65 | Education and algorithm aimed at certificed medical assistants | PPV13 prescriptions increased from 74 prescriptions before the project to 165 presriptions after (no p value). Administration of PPSV23 iproved from 32 immunizations to 55 immunizations (no vaplue). Time frames were not specified (Data was collected over a three-month period). |
|  | Webster et al. (2019), Australia | National cross-sectional surveys before and after vaccination funding initiatives | 11239 Indigenous (Native Aboriginal and Torres Strait Islander) individuals | National funding of pneumococcal vaccination | There was a decline in pneumococcal vaccination coverage for Indigenous adults between 2004-05 and 2012-13. |
|  | Wells et al., (2019), USA | Retrospective single-center cohort, no control group | 84 patients | Pharmacy resident-led vaccination protocol to interview each patient for pneumococcal history and verify medical records, then contact the most responsible healthcare provider | 64.7% of unvaccinated patients were vaccinated prior to discharge following the intervention. |
| III. Primary care interventions | Bitton et al., (2016), USA | Cross-sectional population survey via telephone to determine whether there was an association between primary care and pneumococcal vaccination | 16,731 residents | The measured exposure was individuals with “enhanced primary care”, aka – regular, personal provider who “always” or “almost always” has knowledge of their medical history, providers prompt appointments, was up-to-date regarding specialist care, and asked them about all of their medications. | In the participants who reported indicators of enhanced primary care, there was a higher rate of vaccination (51% versus 43%, no p value reported). |
|  | Bowen et al., (2016), USA | Before-after study of multiple screening parameters, with measures calculated 12 months after implementation | 10,917 patients cared by primary care services | Clinical Decision support tool implemented into Electronic Medical Records, with and without alterations for patient-tailored care | Vaccination rates significantly increased (6.1% to 14.5% absolute change from baseline, p<0.05). |
|  | Burka et al. (2019), USA | Single-center retrospective analysis following intervention, no control, in veterans who had been hospitalized | 540 older adults | Discharge reminder tool within required discharge note to provide patient-specific guidance | Vaccination at discharge in vaccine naïve patients increased from 1.4% to 25% following implementation, however, there were a significant number of inappropriately (i.e. given for the wrong age or target group) given vaccinations (n=20 of 20). |
|  | Church et al. (2018), USA | Before-after design, with primary outcome of administration following 180 days following placement of order | 160 participants with chronic lymphocytic leukemia | Virtual clinic through electronic medical record to identify patients within a chronic lymphocytic leukemia clinic | 100/160 eligible patients had received the recommended vaccine following 180 days. |
|  | Hoang et al. (2018), USA | Population-based analysis of 6-year period through electronic medical record, to analyze the association between annual periodic health exams and receipt of vaccine | 10,318 Medicare beneficiaries in the USA | Receipt of the periodic health exam (i.e. annual physical) during a period where Medicare in the USA did not cover annual physicals (before 2011) | Patients with a physical health exam were more likely to obtain pneumococcal vaccination (p<0.0001, OR 1.10). |
|  | Hurley et al. (2018), USA | Pragmatic randomized controlled trial, measuring documentation of receipt of any vaccination <6 months after the intervention | 5,332 older adults over 65 years of age | Centralized vaccine reminder/recalls using an Immunization Information System | The intervention, while effective for influenza vaccination, was ineffective at increasing pneumococcal rates in any studied population (9.0% versus 8.4%, no p value reported). |
|  | Kilgore et al. (2017), USA | Pre/post assessment across 15 ambulatory care clinics, no control group | Sample size not reported specific to pneumococcal vaccination | Multicomponent intervention including provider education, clinic vaccine champions, electronic health record integration, student pharmacist placement for patient education, provider alerts, messaging patients on a patient portal, insurance claims processing systems | Vaccination increased from 25.7% to 68.4% in the intervention period (no p value reported). |
|  | Loskutova et al. (2020), USA | Prospective interventional before-and-after study, non-randomized, with 43 primary care physicians | 43 providers; 76,503 and 58,948 unique patients across three years in intervention and control groups respectively | Multicomponent intervention with reminders, provider-level performance reports, provider education, patient visual aid materials, standing orders for immunizations | There was no significant difference in pneumococcal vaccination from baseline in the intervention group (35.7 ± 19.6 to 34.5% ± 19.0%, p = 0.3) |
|  | Zimmerman et al. (2016), USA | Cluster randomized control trial across 25 primary care practices | 18,107 older adults | Four Pillars program implemented after patient research including provider education and one-on-one coaching. | There were mixed results related to vaccination uptake, particularly across sites. In small or medium-sized private primary care practices in Pittsburgh, there were significant increases in pneumococcal vaccination while there were no differences in larger safety net practices in Houston. |
|  | Zorek et al., (2015), USA | Before-after pilot study with multiple screening and public health variables analyzed | 34 patients (1 lost to follow-up) | Interprofessional teaching linic to provide preventative health services, including vaccination | 21% increase in pneumococcal vaccines (not significant, p=0.16) following intervention. |

Supplementary Table 2: Risk of Bias Assessment – Cochrane ROB and Cochrane RoBANs

*This tool was applied only to full-text studies that featured a randomized study design. A score of 0 indicates low risk, 1 indicates medium/unclear risk, and 2 indicates high risk. The criterion related to blinding was removed from the assessment tool due to the lack of applicability to the intervention.*

| **Citation, Country** | **Participant Selection (e.g. eligible and clinically relevant for vaccination)** | **Confounding (e.g. adequate control groups or controlling of variables)** | **Measure of Exposure (e.g. length of folow-up, adequate outcomes)** | **Incomplete Outcome Data (e.g. loss to follow-up, lack of reporting)** | **Selective Outcome Reporting (e.g. discrepency between stated goals/outcomes and measurin, incomplete outcome reporting)** | **Overall Assessment** |
| --- | --- | --- | --- | --- | --- | --- |
| Bitton et al., (2016), USA | 0 | 0 | 1 | 0 | 1 | **Low** |
| Bowen et al., (2016), USA | 0 | 2 | 0 | 0 | 1 | **Medium** |
| Brown et al. (2016), USA | 0 | 2 | 1 | 0 | 1 | **Medium** |
| Burka et al. (2019), USA | 0 | 2 | 1 | 0 | 1 | **Medium** |
| Caffrey et al. (2017), USA | 0 | 2 | 1 | 0 | 1 | **Medium** |
| Carillo et al. (2017), USA | 0 | 2 | 1 | 0 | 1 | **Medium** |
| Chan et al., (2015), Hong Kong | 0 | 0 | 1 | 0 | 0 | **Low** |
| Church et al. (2018), USA | 0 | 2 | 1 | 0 | 1 | **Medium** |
| Ho et al. (2017), Singapore | 0 | 2 | 1 | 0 | 1 | **Medium** |
| Ho et al. (2019, Singapore | 0 | 0 | 1 | 0 | 0 | **Low** |
| Hoang et al. (2018), USA | 0 | 2 | 1 | 0 | 1 | **Medium** |
| Hurley et al. (2018), USA | 0 | 0 | 1 | 0 | 0 | **Low** |
| Jolin et al., (2016), USA | 0 | 2 | 1 | 0 | 1 | **Medium** |
| Kawczak et al., (2020), USA | 0 | 0 | 1 | 0 | 1 | **Low** |
| King, Judd et al. (2017), USA | 0 | 2 | 0 | 0 | 1 | **Medium** |
| Loskutova et al. (2020), USA | 0 | 0 | 1 | 0 | 1 | **Low** |
| McAdam-Marx et al. (2020), USA | 0 | 2 | 1 | 0 | 1 | **Medium** |
| McKeirnan and Sarchet et al. (2019), USA | 0 | 2 | 0 | 0 | 1 | **Medium** |
| Mendu et al. (2014), USA | 0 | 2 | 0 | 0 | 1 | **Medium** |
| Naito et al. (2020), Japan | 0 | 2 | 1 | 0 | 1 | **Medium** |
| Patel et al., (2018) USA | 0 | 2 | 0 | 0 | 1 | **Medium** |
| Pickren et al. (2016), USA | 0 | 2 | 0 | 0 | 1 | **Medium** |
| Pizzi et al. (2018), USA | 0 | 0 | 0 | 0 | 1 | **Low** |
| Shono et al. (2019), Japan | 0 | 0 | 1 | 0 | 1 | **Low** |
| Turbeville et al. (2018), USA | 0 | 2 | 1 | 0 | 1 | **Medium** |
| Webster et al. (2019), Australia | 0 | 2 | 1 | 0 | 1 | **Medium** |
| Wells et al., (2019), USA | 0 | 0 | 0 | 0 | 1 | **Low** |
| Zimmerman et al. (2016), USA | 0 | 2 | 1 | 0 | 1 | **Medium** |
| Zorek et al., (2015), USA | 0 | 2 | 0 | 0 | 1 | **Low** |
